# Moisture-Driven Morphology Changes in the Thermal and Dielectric Properties of TPU-Based Syntactic Foams

**DOI:** 10.3390/polym17050691

**Published:** 2025-03-05

**Authors:** Sabarinathan Pushparaj Subramaniyan, Partha Pratim Das, Rassel Raihan, Pavana Prabhakar

**Affiliations:** 1 Department of Mechanical Engineering, University of Wisconsin-Madison, Madison, WI 53706, USA; pushparajsub@wisc.edu; 2 Department of Mechanical and Aerospace Engineering, University of Texas at Arlington, Arlington, TX 76019, USA; parthapratim.das@mavs.uta.edu (P.P.D.); mdrassel.raihan@uta.edu (R.R.); 3 Department of Civil & Environmental Engineering, University of Wisconsin-Madison, Madison, WI 53706, USA

**Keywords:** thermoplastic polyurethane (TPU), syntactic foams, moisture aging, thermal transport and dielectric, microphase morphology

## Abstract

Syntactic foams are a promising candidate for applications in marine, oil and gas industries in underwater cables and pipelines due to their excellent insulation properties. The effective transmission of electrical energy through cables requires insulation materials with a low loss factor and low dielectric constant. Similarly, in transporting fluid through pipelines, thermal insulation is crucial. However, both applications are susceptible to potential environmental degradation from moisture exposure, which can significantly impact the material’s properties. This study addresses the knowledge gap by examining the implications of prolonged moisture exposure on thermoplastic polyurethane elastomer (TPU) and TPU-derived syntactic foam via various multi-scale material characterization methods. This research investigates a flexible syntactic foam composed of TPU and glass microballoons (GMBs) fabricated through selective laser sintering. The study specifically examines the effects of moisture exposure over periods of 90 and 160 days, in conjunction with varying GMB volume fractions of 0%, 20%, and 40%. It aims to elucidate the resulting microphase morphological changes, their underlying mechanisms, and the subsequent impact on thermal transport and dielectric properties, all in comparison to unaged samples of the same material. Our findings reveal that increasing the volume fraction of GMB in TPU-based syntactic foam reduces its thermal conductivity and specific heat capacity. However, moisture exposure did not significantly affect the foam’s thermal conductivity. Additionally, we found that the dielectric constant of the syntactic foams decreases with increasing volume fraction of GMB and decreasing frequency of the applied field, which is due to limited molecular orientation in response to the field. Finally, moisture exposure affects the dielectric loss factor of TPU-based syntactic foams with GMBs, possibly due to the distribution morphology of hard and soft segments in TPU.

## 1. Introduction

Block copolymers, known as thermoplastic polyurethane elastomers (TPUs), are composed of alternating hard and soft segments, with the soft segments typically derived from polyester or polyether (polyol) and the hard segments produced by combining a chain extender with an isocyanate. The incompatibility between the soft and hard segments can cause microphase separation. Although these separation structures are observed at the nanoscale, the older term of microphase morphology will be used. Alterations in the TPU’s microphase morphology play a crucial role in determining the material’s functional properties [1,2]. By varying the formulation and constitution, as well as incorporating additives, the mechanical properties of TPUs, including modulus, strength, hardness, damping, and tribological performance, can be customized. TPUs are incredibly versatile, with widespread use in various applications such as sealings, hoses, shoe soles, cable sheaths, films, foams, and automotive interiors. They exhibit the processing abilities of plastics and the high elasticity of rubber, making them a sought-after material in the industry. Moreover, TPUs are well known for their low-temperature flexibility, excellent abrasion resistance, good processing characteristics, and biocompatibility, making them ideal for use in diverse fields, including transportation, construction, and biomedical materials [3,4,5,6,7].

Considerable research has been carried out to investigate the impact of hard-to-soft segment ratio, hard segment type, chain extender type, and segment length on various properties of thermoplastic polyurethane elastomer (TPU). Hydrogen bonding, crystallization behavior, mechanical performance, thermal transport, radiation stability, and dielectric properties of TPU have been extensively studied [8,9,10,11,12,13]. Numerous studies have been conducted on the impact of moisture on TPU, with a notable emphasis on mechanical degradation [7,14,15,16,17,18,19,20]. Despite the large volume of research that has been conducted on TPU, a significant knowledge gap remains regarding the long-term effects of moisture-induced microphase morphology changes caused by hydrolysis, and specifically how these changes affect thermal transport and dielectric properties. Our current study aims to bridge this gap by establishing a correlation between microphase morphology and its properties.

Syntactic foams are a type of closed-cell foam composed of hollow microspheres that are embedded within a matrix material. The microspheres themselves are typically constructed from materials such as glass, cenosphere (a waste material produced by the combustion of fly ash), or metal, while the matrix materials are typically composed of polymers, ceramics, or metals. Among the various types of syntactic foams, polymer-based foams are particularly popular in a range of different industries, including the marine, automotive, aerospace, and electrical sectors. This is largely due to the fact that they offer numerous desirable properties, such as high strength, low density, high buoyancy, low thermal expansion, vibrational damping, and acoustic, thermal, and electrical insulation. These properties make them ideal for a wide range of applications, including energy-absorbing cores in sandwich composites, radome material, acoustic insulation for underwater sonar devices, thermal insulation, and electrical cable insulation. Importantly, the properties of syntactic foams can be customized by altering the matrix type, the volume fraction of the hollow microspheres, and the thickness of the hollow microspheres’ walls [21,22,23,24,25]. In the present work, thermoplastic polyurethane elastomer was used as the matrix material in conjunction with soda lime borosilicate-based glass microballoon reinforcements.

Elastomeric and rubber-based syntactic foams have been the subject of significant advancements in recent years, with researchers developing materials that exhibit remarkable versatility across a range of industries. Notably, these foams have been employed in the design of shoe soles, pneumatic tires, wires, and cable compounds. Furthermore, recent research has demonstrated the immense potential of thermoplastic polyolefin elastomer-based syntactic foam for buoyancy and coaxial cable insulation [26]. Tripathi et al. [27] developed a fire-protective clothing material comprising flexible silicone hollow glass microballoons syntactic foam. Their investigation revealed that applying syntactic foam coating on the glass fabric resulted in a notable improvement, with a second-degree burn time of 10 s, compared to the 6.4 s observed for the glass fabric alone. In the current study, TPU reinforced with glass microballoons (GMBs) was manufactured using selective laser sintering (SLS).

In our prior study [28], we conducted a deep dive into understanding the role of GMB reinforcement and moisture aging on the degradation mechanisms in the viscoelastic properties of TPU-based syntactic foams produced using the SLS process [29]. The existing literature on TPU-based composites produced via SLS has predominantly focused on boosting thermal and electrical conduction properties [30,31,32,33,34,35,36]—consequently, a gap in the realm of thermal and electrical insulation warrants further investigation.

The primary aim of this study is to explicate the impact of internal structure and moisture-triggered deterioration on the functional characteristics of TPU-based syntactic foams fabricated through additive manufacturing. Furthermore, this paper investigates how prolonged exposure to moisture alters the microphase morphology and chemical composition of TPU and TPU-based syntactic foams and the consequential effects on their thermal transport and dielectric properties.

## 2. Methodology

### 2.1. Manufacturing and Conditioning

In this study, we examine syntactic foams that are fabricated from a thermoplastic polyurethane elastomer matrix (FLEXA Grey–Sinterit) and hollow micro-glass balloon fillers (K20–3M), which are produced through the process of selective laser sintering. To prepare the composite powder, TPU and GMBs are mixed in different volume ratios (20% and 40%) using an automated mixer. The mixing process involves using the mixer for 5 min at 30 V and 3 min at 70 V to ensure proper dispersion of GMBs in TPU. Once mixed, the powder is then fed into the SLS equipment (Lisa-pro) for sintering, with a layer height of 0.075 mm and an input laser power ratio of 1.5. The input parameters are determined based on a parametric study conducted by Tewani et al. [29]. The resulting samples, including pristine TPU and TPU with 20% and 40% GMBs, are then immersed in de-ionized water at 23 °C. Afterward, they are removed and desorbed at 50 °C in an oven for 24 h to eliminate any free water present in the samples. The primary objective of this study is to gain insights into the effect of bound water on the thermal and dielectric properties of the samples.

### 2.2. Thermal Transport Characterization

**Figure 1:**
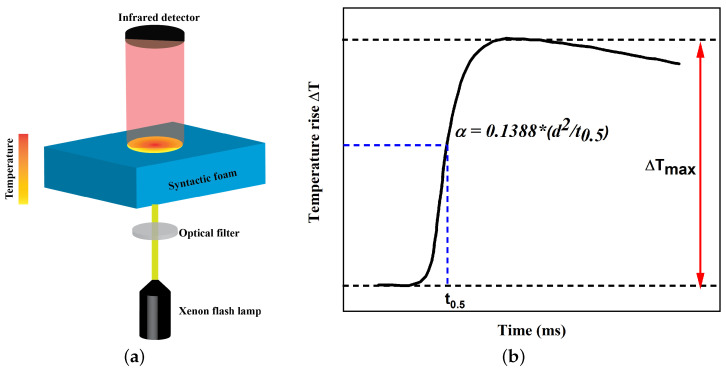
(**a**) Schematic illustration of laser flash analysis and (**b**) thermal diffusivity measurement using time vs. temperature rise plot.

Laser flash analysis (LFA) is a widely used thermal analysis technique to measure thermal transport properties. It involves using a high-intensity laser to generate a short pulse of heat that is applied to a small spot on the rear surface of a sample. The resulting temperature rise on the front surface is measured using a detector as shown in Figure 1a. Later, the thermal diffusivity is calculated from the temperature rise signal shown in Figure 1b. In this work, the thermal diffusivity of TPU and TPU-based syntactic foam is measured using NETZSCH LFA 447 (Netzsch, Selb, Germany) over a temperature range from 25 °C to 100 °C. For the purposes of this investigation, a specimen with dimensions of 10 × 10 × 1 mm^3^ is chosen, and the half rise time is measured using the Radiation+pulse model in Netzsch proteus analysis software.

(1) $$\alpha =0.1388*\frac{d^{2}}{t_{0.5}}$$

where α, *d*, and $t_{0.5}$ refer to thermal diffusivity in mm^2^/s, sample thickness in mm, and half raise time, as shown in Figure 1b. The specific heat of the samples is measured using DSC, and the measured density of syntactic foams is input into Equation (2) to obtain the thermal conductivity κ:

(2) $$\kappa =\alpha *\rho *C_{p}$$

### 2.3. Dielectric Characterization

The dielectric properties of the syntactic foam are measured using Novocontrol Broadband Dielectric Spectroscopy assisted with an alpha analyzer to measure the complex dielectric and impedance properties of syntactic foams as a function of frequency. The dielectric properties are measured at room temperature by utilizing the parallel plate capacitor, where 1000 mV of voltage is applied across a sample size of 30 mm diameter with 1 mm thickness at frequencies ranging from 10 Hz to 10^6^ Hz. The frequency range under consideration encompasses valuable information regarding molecular and dipolar fluctuations. Furthermore, charge transport and polarization effects, which manifest at both the inner and outer boundaries, can be effectively captured through dielectric properties [37]. The dielectric values are obtained by analyzing the phase shift between the applied voltage and the current response. The real and imaginary parts of complex dielectric are called dielectric constant and dielectric loss, respectively. tanϕ is the dielectric loss tangent.

## 3. Results

### 3.1. Microphase Morphological Characterization of TPU-Based Syntactic Foam

**Figure 2:**
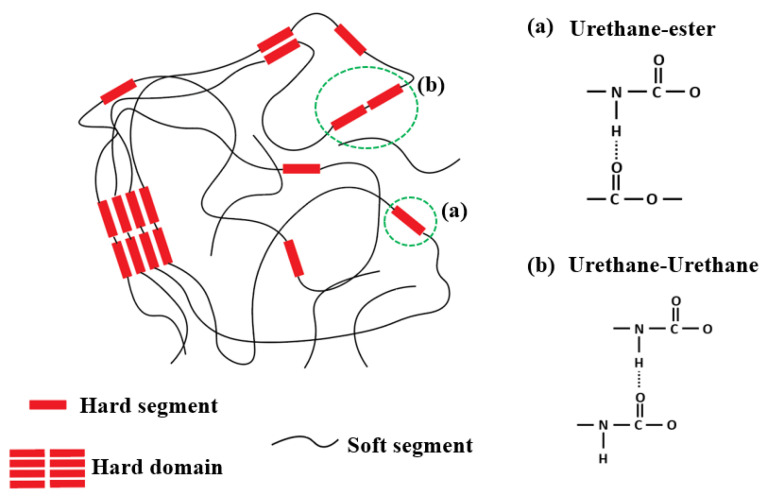
Graphical illustration of the hydrogen bonding present in thermoplastic polyurethane elastomer. (Reprinted with permission from [28], Copyright 2023, Elsevier).

In our previous research [28], we investigated the microphase morphological changes in TPU-based syntactic foams exposed to moisture, utilizing chemical and thermal characterization methods on both unaged and aged samples. For the chemical characterization aimed at analyzing microphase morphology, Fourier Transform Infrared Spectroscopy (FTIR) was employed to determine the Degree of Phase Separation (DPS) and the Degree of Phase Mixing (DPM), based on the hydrogen bonding present in the carbonyl groups. Figure 2 illustrates various types of hydrogen bonding in TPU. A higher DPS indicates an increased formation of inter-urethane bonds (hard-hard segment), whereas a higher DPM signifies greater cohesion between the hard and soft segments.

**Figure 3:**
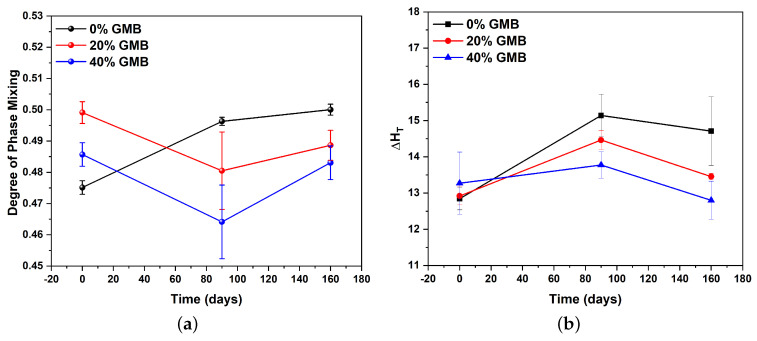
(**a**) Degree of phase mixing as a function of GMB volume fraction and moisture exposure duration. (**b**) The total melting enthalpy for different GMB volume fractions and moisture exposure times. (Reprinted with permission from [28], Copyright 2023, Elsevier).

Figure 3a shows that the pristine samples of TPU reinforced with 20% GMBs have higher DPM, followed by 40% GMBs, and then neat TPU. After 90 days of moisture exposure, only neat TPU showed an increase in DPM, whereas the other samples showed a reduction. However, after 160 days of moisture aging, the DPM increased with an increase in the volume fraction of GMBs, while neat TPU showed only a marginal increase. This could be attributed to water molecules forming bridges between NH−C=O and two C=O groups, which weakens the original hydrogen bonds. When the water molecules desorb, the polymer’s interaction with them decreases, and the original hydrogen bonding is restored [14]. However, after 160 days of moisture exposure, the ester hydrolysis in the soft segment becomes prominent. This leads to chain scission, which increases DPM and decreases DPS. A recent study conducted by Yang et al. [38] demonstrates that the hydrolysis of esters is expedited by acidic molecules generated during prolonged exposure to moisture. Furthermore, as hydrolytic degradation advances, the tightly packed hard segments are adversely impacted by the intrusion of water, which supports our experimental findings. Similar behavior was observed by Jouibari et al. [39], where the DPS increased with a longer soft segment.

**Figure 4:**
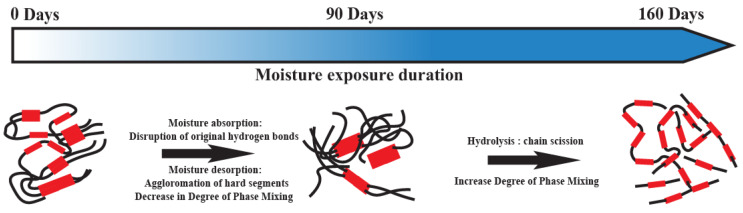
Illustration of moisture-induced mechanisms in TPU elastomer at different times of exposure.

The analysis of microphase morphology through thermal characterization involves the calculation of the area under the melting enthalpy peak. TPU exhibits a broad transition peak with multiple underlying melting enthalpy peaks, each corresponding to different hard segment morphologies. A higher melting enthalpy indicates a greater quantity of inter-urethane bonds, specifically hard segment-hard segment bonds since it requires more energy to break those bonds. Figure 3b demonstrates that the total melting enthalpy of all samples increased significantly after 90 days of moisture exposure. This increase is primarily due to the formation of more hard domains. Conversely, after 160 days of moisture exposure, the total melting enthalpy decreases. This decrease occurs because the hydrolysis of the soft segment predominates, causing chain scission of long-chain polyol at an accelerated rate [18]. As a result, more short-chain diol is produced, which subsequently forms more hard–soft segment bonds, distributing homogeneously throughout the TPU system. These mechanisms are illustrated in Figure 4. The methodology for calculating microphase morphology and the corresponding results are comprehensively detailed in our previous work [28].

### 3.2. Influence of Moisture and GMB Volume Fraction on Thermal Transport Properties of Syntactic Foam

**Figure 5:**
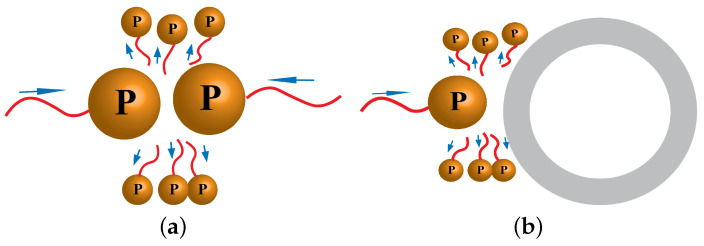
(**a**) Phonon–phonon scattering and (**b**) phonon–boundary scattering.

Phonons are discrete units of energy generated by atomic lattice vibrations, which are responsible for heat transfer in insulating materials such as polymers. The thermal conductivity of polymer and polymer composite materials relies on the average distance phonons travel before experiencing scattering, known as the mean free path. A higher mean free path results in higher thermal conductivity. Three distinct types of scattering, namely phonon–phonon scattering, phonon–boundary scattering, and phonon–impurity scattering, influence the phonon mean free path. Temperature influences the phonon’s mean free path, where the wavelength of the dominant phonon decreases with an increase in temperature, which means the phonon wavelength is higher at low temperatures. As the temperature increases, the phonon’s mean free path decreases due to phonon–phonon scattering as illustrated in Figure 5a, consequently reducing the thermal conductivity. At low temperatures, the long wavelength phonons are not affected by defects or impurities as they are at the atomic scale. Hence, the phonon at low temperatures majorly experiences phonon–boundary scattering as illustrated in Figure 5b. However, as the temperature increases, the dominant phonon’s wavelength becomes comparable to defects and impurities, and phonon–impurity scattering becomes active. In contrast, further increase makes the phonon wavelength a size comparable to the crystal size, so phonon–phonon scattering becomes dominant [40,41].

With an increase in the volume fraction of GMBs in TPU, both the thermal conductivity and specific heat capacity were observed to decrease. For instance, at a temperature of 25 °C, it was found that the thermal conductivity of unaged TPU with 20% GMBs and 40% GMBs decreased by 38.42% and 56.98%, respectively, when compared with unaged neat TPU. The decrease in thermal conductivity with an increase in the volume fraction of GMBs can be attributed to the increase in void contents in the TPU. The thermal conductivity of the K20 GMBs utilized in this study is considerably low, measuring only 0.070 W/mK at 25 °C. Furthermore, an increase in the volume fraction of GMBs also decreases the specific heat capacity. Specifically, the specific heat capacity of a material pertains to the amount of energy required to raise the temperature of 1 g of a material by one degree Celsius. Neat TPU exhibits a tendency to absorb more energy in order to increase its temperature, whereas an increase in the volume fraction of GMBs reduces the total amount of polymer, subsequently leading to a decrease in the specific heat capacity.

**Figure 6:**
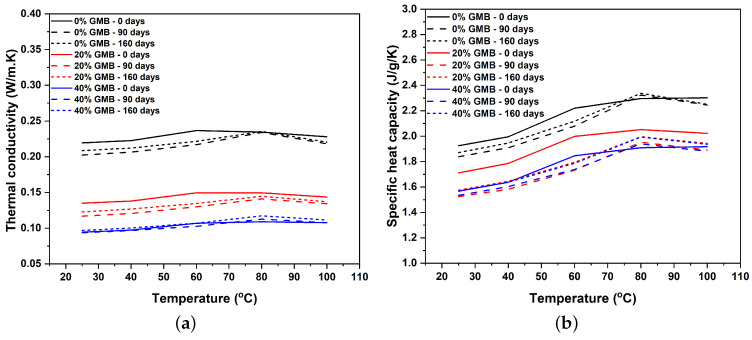
(**a**) Thermal conductivity and (**b**) specific heat capacity of TPU with different volume fractions of GMB reinforcement and moisture exposure time.

The thermal conductivity of the polymer is also dependent on the exposure temperature since the polymer undergoes significant structural changes like glassy below the glass transition temperature and leathery and rubbery above the glass transition temperature and finally reaching the terminal region. In this work, TPU is an elastomer with a multiblock structure with different phase-separated morphology that shows complex thermal transport behavior as the temperature changes. So, we opted for a temperature range between 25 °C and 100 °C. Figure 6a shows that the thermal conductivity of TPU and TPU-based syntactic foams did not change significantly with temperature. However, around 60 °C, there was a slight increase in the thermal conductivity, with marginal reduction until 100 °C. For unaged samples, this rise in thermal conductivity is associated with the melting of short-order crystallites. One main reason for this plateau behavior initially is due to the amorphous nature of the soft segment in TPU. As the temperature increases, the main chain movements will create more microvoids, enhancing the phonon scattering and decreasing the thermal conductivity. At the same time, there is an opposing reaction where the dominant chain movements might increase chain alignments and bring the chain arrays closer, increasing the thermal conductivity and eventually resulting in a plateau over a specific temperature [42]. Correspondingly, the material’s specific heat capacity increases at elevated temperatures, primarily attributable to heightened kinetic energy manifested from atomic motion and augmented potential energy associated with interatomic bond distortions. The specific heat capacity of unaged specimens, similar to thermal conductivity, exhibits an abrupt shift in behavior at approximately 60 °C, which is attributed to the melting of short-ordered crystallites. In contrast, all aged specimens demonstrate a notable upward shift to 80 °C. This change is due to prolonged exposure to moisture, which creates both more ordered and disordered long-range hard segments through the movement of the short-ordered crystallites found in unaged samples. The melting of these long-range segments requires more energy, thereby increasing their specific heat and thermal conductivity. This finding is consistent with the results discussed in Section 3.1. When assessing the thermal conductivity and specific heat capacity of a 90-day moisture-aged sample compared to a 160-day moisture-aged sample, no significant variations were observed that warrant substantial commentary. The specific heat capacity exhibits a more pronounced decrease for the 20% GMB samples when compared to the other concentrations, in relation to their initial values at day 0. This observation is attributable to the fact that at day 0, the ratio of hard segment morphologies to the overall TPU present in the TPU-based syntactic foam is substantially higher in the 40% GMB samples. However, following a 90-day period of moisture exposure, this ratio undergoes a significant shift, becoming more prominent in the 20% GMB samples. This alteration occurs because the 90-day moisture exposure does not markedly affect the 40% GMB samples, which inherently possess a higher concentration of hard domains. This phenomenon can be observed in Section 3.1. However, a slight decline was noted in thermal conductivity and specific heat capacity upon comparing the unaged sample with the aged samples of the same material type. This reduction may be attributed to increased phonon scattering resulting from a higher inhomogeneous distribution of hard and soft domains and increased interphase resistance in the 90-day- and 160-day-aged samples.

### 3.3. Influence of Moisture and GMB Volume Fraction on Dielectric Properties of Syntactic Foam

**Figure 7:**
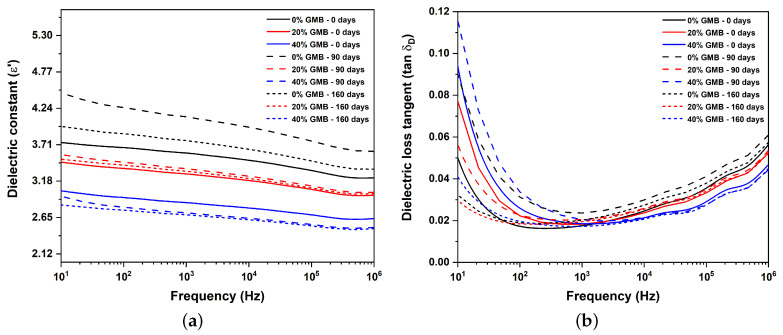
Moisture-driven changes in dielectric properties as a function of GMB volume fraction and frequency: (**a**) dielectric constant and (**b**) dielectric loss tangent.

**Figure A1:**
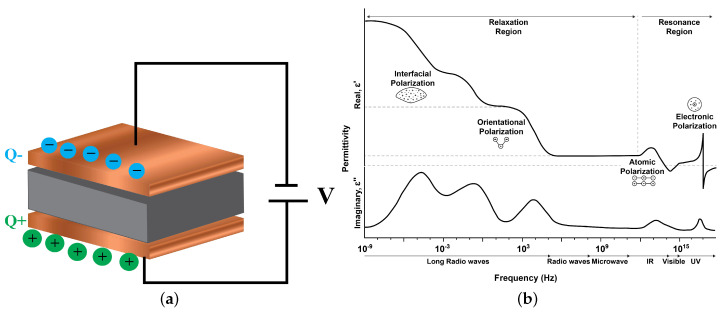
(**a**) Schematic illustration of dielectric analysis and (**b**) dielectric response and attributing mechanism at different frequencies [50].

The dielectric constant explains the material’s ability to store electrical charge through different types of polarization mechanisms (i.e., electronic, ionic, atomic, orientational, and interfacial) when we apply an electric field, which is achieved by the chemical compounds in polymers. All the dielectric mechanism can be found in Appendix A Figure A1. The data depicted in Figure 7a demonstrate that increasing the volume fraction of GMBs reduces the material’s dielectric constant. This phenomenon can be ascribed to the decline in the number of polymer chains participating in the polarization mechanism as the quantity of GMBs increases. It is important to note that GMBs are hollow particles with a thin soda lime borosilicate glass covering, and it is commonly accepted that the dielectric constant of air is 1. A similar trend is also seen by other researchers [43,44,45]. The frequency domain dielectric constant response in all the samples shows a similar trend, as observed in Figure 7a. A higher dielectric constant is evident in the lower frequency range (<100 Hz). This behavior is attributed to Maxwell-Wagner-Sillars polarization or interfacial polarization in all heterogeneous dielectric materials [46,47,48]. TPU, in this study, is a block copolymer with a multi-phase system where interfacial polarization occurs between hard and soft segments due to different dielectric constants, as reported in [11,13]. The orientational polarization phenomenon also contributes to the higher dielectric constant in the lower frequency range (10–1000 Hz). In this range, the soft and hard segments have sufficient time to orient themselves in response to an applied alternating electric field. This leads to easy mobility of molecules for orientational polarization, resulting in a high dielectric constant, as illustrated in Figure 7a.

During the comparison of the change in dielectric constant for all samples at varying moisture exposure times, it was observed that the neat aged TPU showed a significant increase in dielectric constant across measured frequencies compared to unaged samples. However, after 160 days of moisture exposure, the dielectric constant decreased compared to 90 days but was still slightly higher compared to unaged TPU. A similar trend was noticed in the TPU with 20% GMBs. However, in the case of TPU with 40% GMBs, the dielectric constant decreased with an increase in the exposure time. One significant factor contributing to the observed trend is that neat TPU tends to have a higher concentration of chemical compounds that can participate in polarization. After 90 days of moisture exposure, distinct morphologies of hard segments are formed, as discussed in Section 3.1. These hard segments are highly polar (–NH–C=O) in nature and can easily undergo orientational polarization when an alternating field is applied. A similar trend is also observed in TPU with 20% GMBs. However, for TPU with 40% GMBs, the dielectric constant decreases after 90 days of exposure. This is because the GMB density per unit volume is higher in this specimen, which hinders the orientational polarization of the hard segments in the applied alternating electric field.

The dissipation factor, also called the loss tangent or tan delta, serves as a metric for quantifying energy loss or dissipation in dielectric materials under the influence of an alternating field. This dimensionless quantity is defined as the ratio of the imaginary part of permittivity to the real part of permittivity, and it measures the efficiency of dielectric materials without significant losses. A lower dissipation factor denotes a higher efficiency and superior performance of dielectric materials. However, several factors, such as molecular interactions, defects, and electrode interfaces, impede dipole alignment, which takes time to occur. Consequently, there is a delay between dipole orientation and the applied alternating field, leading to energy losses in the form of heat.

Figure 7b shows the dielectric loss tangent (dissipation factor) for unaged and aged samples with different GMB volume fractions. It can be seen from the figure that the dissipation factor of unaged samples is higher for a higher volume fraction of GMBs at 10 Hz frequency, which is attributed to interfacial polarization. Interfaces are formed when a filler or multiphase material is put together. The interfacial polarization is prominent at lower frequencies, resulting in a higher loss due to interface friction or interfacial polarization. The interfacial polarization becomes less dominant at higher frequencies, resulting in less loss and a lower dissipation factor. However, after $10^{3}$ Hz, there is a reversal in trend, with the dissipation factor being highest for neat TPU, followed by TPU with 20% and 40% GMBs. This trend continues up to $10^{6}$ Hz. All specimens exhibit a slight increase in dissipation factor with increasing frequency beyond $10^{3}$ Hz. At higher frequencies, the dissipation factor reaches its highest value for the neat TPU sample, followed by TPU samples containing 20% and 40% GMBs, respectively. The primary factor contributing to this behavior is the occurrence of leakage loss. It is important to note that a combination of relaxation polarization loss and leakage loss influences the dissipation factor [49]. Consequently, an increase in the material’s electrical conductivity leads to an escalation in leakage loss. The impact of leakage loss becomes more pronounced at higher frequencies due to the rapid alteration in the electric field, which prevents the molecules from aligning and adequately accommodating these charges, resulting in subsequent leakage.

After a 90-day exposure to moisture, it is observed that the dissipation factor of TPU containing 40% GMBs surpasses that of neat TPU and TPU with 20% GMBs at a frequency of 10 Hz. However, at a frequency of $10^{3}$ Hz, the dissipation factor of neat TPU exceeds that of TPU with 20% and 40% GMBs. This trend persists in samples exposed to moisture for 160 days as well. The primary factors contributing to these observations are the combined effects of leakage and interfacial polarization loss. Specifically, at 10 Hz and after 90 days of moisture exposure, the TPU exhibits higher electrical conductivity, followed by TPU with 40% GMBs and TPU with 20% GMBs. Simultaneously, a higher volume fraction of GMBs enhances interfacial polarization loss. Consequently, TPU with 40% GMBs demonstrates a higher dissipation factor, followed by neat TPU and TPU with 20% GMBs. A similar trend is observed after 160 days of moisture exposure, albeit with significantly lower dissipation factor values. This can primarily be attributed to the confinement of highly polar hard segments within the soft domain, preventing the polarization of molecules under the applied electric field.

## 4. Conclusions

**Figure 8:**
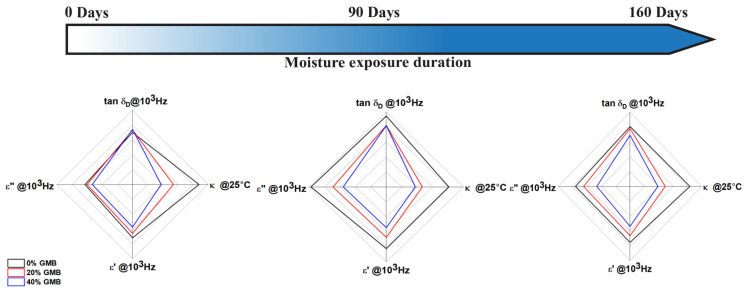
Summary of thermal and dielectric property evolution with exposure times.

This study sought to investigate the long-term degradation caused by moisture in TPU and TPU-based syntactic foams, as well as the accompanying changes in their microphase morphology. In order to achieve this objective, the samples were submerged in water and subjected to testing at two distinct time intervals: 90 days and 160 days. Furthermore, this study sought to elucidate the functional implications of these alterations in thermal and dielectric characteristics, and the diagrams illustrating the change in these characteristics are outlined in Figure 8.

### 4.1. Thermal Transport

The TPU-based syntactic foam’s thermal conductivity and specific heat capacity decrease as the volume fraction of GMBs increases from 0% GMBs to 20% GMBs and 40%. For each of these volume fractions, the thermal conductivity remains relatively stable with temperature variations up to 100 °C, except for a sharp increase around 60 °C, corresponding to the melting of short-ordered crystallites. Similarly, the specific heat capacity generally increases with temperature but exhibits a noticeable change around 60 °C. In the case of moisture-exposed samples, this change shifts to higher temperatures due to moisture-induced dissociation and reorganization of hard segments. At room temperature (25 °C), it was observed that the thermal conductivity of 40% GMB-reinforced syntactic foam exposed to moisture for 90 days had a negigible change compared to the unaged values. Further, the difference in thermal conductivity for all foams between the 90-day and 160-day moisture exposure was negligible.

### 4.2. Dielectric Properties

The dielectric constant decreases as the volume fraction of GMBs increases. Additionally, it follows a decreasing trend as the frequency of the applied field increases. This can be attributed to the limited time available for the molecules to orient themselves in response to the applied field, consequently reducing the material’s dielectric constant. It is important to note that after 160 days of moisture exposure, there was a 35.4% reduction in the dielectric loss factor of neat TPU at 10 Hz compared to unaged neat TPU, and a 1.86% reduction at $10^{6}$ Hz. However, there was a 79.5% increase in the dielectric loss factor in neat TPU exposed to moisture for 90 days at 10 Hz, and an 8.20% increase in the dielectric loss factor at $10^{6}$ Hz. It is noteworthy that similar behavior is also observed in TPU-based syntactic foams with 20% and 40% GMBs when compared to their unaged counterparts. One possible reason is the distribution morphology of the hard and soft segments in TPU (GMBs are unaffected by moisture). At 90 days, the disordered hard domains are more prevalent, whereas after 160 days of moisture exposure, the hard segments are uniformly distributed, and the hard domain content is decreased.

The investigation of the thermal transport and dielectric behavior of TPU-based syntactic foam serves a dual purpose: it enhances our understanding of the moisture-induced degradation mechanisms and provides valuable insights into the geometrical hierarchy at various length scales that can be customized to attain desired properties. For instance, at the nanoscale level, the microphase morphology exhibits increased disordered hard domains, leading to an increase in the dielectric constant and dielectric loss. Similarly, when the soft segment length is reduced, it promotes the homogeneous distribution of hard segments and reduces the hard domain region, resulting in a decrease in dielectric loss. At the microscale level, the GMB content can be adjusted to modify the void content, which directly impacts the thermal transport, and dielectric properties. The additive manufacturing technique used in this study offers an added advantage, as it enables the fabrication of tunable materials at the macroscale level.

## Figures and Tables

**Figure A2:**
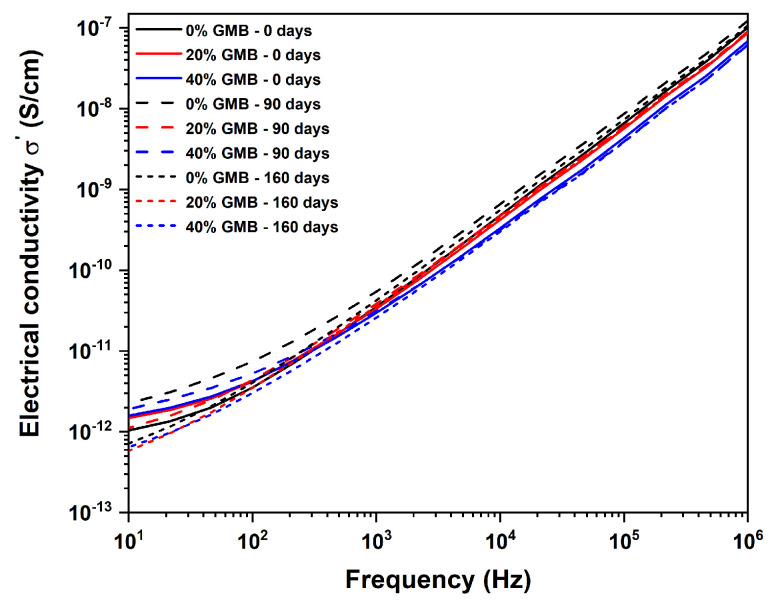
Electrical conductivity of TPU and TPU−based syntactic foam as a function of moisture aging time and frequency.

## Data Availability

Dataset available on request from the authors.

## Appendix A

### Appendix A.1. Dielectric Study

The dielectric mechanisms occurring across a broad spectrum of frequencies are depicted in Figure A1. In the present study, the analyzed frequency spectrum manifests two distinct polarization phenomena. The first pertains to space charge or interfacial polarization, while the second corresponds to orientational or dipolar polarization. Conversely, atomic and electronic polarization phenomena are observed at significantly higher frequencies, as visually represented in Figure A1.

**Orientation or dipolar polarization:** Polymers are composed of various molecules that arise from the combination of atoms, resulting in the sharing of electrons. This sharing of electrons leads to an uneven charge distribution, resulting in permanent dipole moments that are randomly arranged in the absence of an electric field. However, applying an electric field results in torque exertion on the dipole, causing it to rotate and eventually align with the electric field. This alignment results in orientational polarization, which, in turn, leads to the occurrence of friction and dielectric losses [51].

**Space charge or orientational polarization:** If a low-frequency electric field is applied, charge carriers may traverse through the material over a given distance. This movement of migrating charges can be obstructed, leading to interfacial or space charge polarization. The charges may be confined to the interfaces of a material if they are unable to be freely discharged or replaced at the electrodes. This results in a distortion of the electric field, which leads to an increase in the material’s overall capacitance and, consequently, an increase in the dielectric constant. At low frequencies, the charges have sufficient time to accumulate at the borders of the conducting regions, leading to an increase in $\epsilon^{\prime }$. However, at higher frequencies, the charge displacement is relatively small compared to the dimensions of the conducting region, and therefore, polarization does not occur [51].

In the context of dielectric measurement principles, the experimental setup involves positioning the sample within a simple capacitor formed by two electrodes. Subsequently, a voltage denoted as $U_{0}$, operating at a fixed frequency of ω/2π, is applied to the sample, resulting in a corresponding current Io at the identical frequency. Notably, the system response exhibits a phase shift or phase lag, characterized by the phase angle ϕ, representing the temporal difference between the voltage and current waveforms. $U^{*}$, $I^{*}$, $Z^{*}$, $\epsilon^{*}$, and tanϕ denote the complex voltage, complex current, complex impedance, complex permittivity, and dielectric loss factor, respectively.

(A1) $$\begin{array}{c} U\left(t\right)=U_{0}cos\left(\omega t\right)\\ I\left(t\right)=I_{0}cos\left(\omega t+\phi \right) \end{array}$$

(A2) $$\begin{array}{c} U^{*}=U^{\prime }+iU^{\prime \prime }\\ I^{*}=I^{\prime }+iI^{\prime \prime } \end{array}$$

(A3) $$\begin{array}{c} U^{\prime }=U_{0}\\ U^{\prime \prime }=0\\ I^{\prime }=I_{0}cos\phi \\ I^{\prime \prime }=I_{0}sin\phi \end{array}$$

(A4) $$tan\phi =\frac{I^{\prime \prime }}{I^{\prime }}$$

(A5) $$Z^{*}=\frac{U^{*}}{I^{*}}$$

Dielectric properties of the material are related to the impedance measured using Equation (A5), where $C_{0}$ is the capacitance of the empty sample capacitor.

(A6) $$\epsilon^{*}=\epsilon^{\prime }-i\epsilon^{\prime \prime }=\frac{-i}{\omega Z^{*}\left(\omega \right)}\frac{1}{C_{0}}$$
